# Deacetylphylloketal, a New Phylloketal Derivative from a Marine Sponge, Genus *Phyllospongia*, with Potent Anti-Inflammatory Activity in In Vitro Co-Culture Model of Intestine

**DOI:** 10.3390/md17110634

**Published:** 2019-11-08

**Authors:** Seon Min Lee, Na-Hyun Kim, Sangbum Lee, Yun Na Kim, Jeong Doo Heo, Eun Ju Jeong, Jung-Rae Rho

**Affiliations:** 1 Gyeongnam Department of Environment & Toxicology, Korea Institute of Toxicology, 17 Jegok-gil, Munsan-eup 52834, Korea; smlee84@kitox.re.kr (S.M.L.); nhkim@kitox.re.kr (N.-H.K.); jdher@kitox.re.kr (J.D.H.); 2 Department of Oceanography, Kunsan National University, Kunsan 54150, Korea; sblee08@kunsan.ac.kr; 3 Department of Agronomy and Medicinal Plant Resources, Gyeongnam National University of Science and Technology, Jinju 52725, Korea; skdbssk@hanmail.net

**Keywords:** deacetylphylloketal, phyllohemiketal A, *Phyllospongia*, co-culture, intestine, inflammation, inflammatory bowel disease

## Abstract

The inflammatory bowel diseases (IBD) cause chronic inflammation of the gastrointestinal tract and include ulcerative colitis (UC) and Crohn’s disease (CD). The prevalence of IBD has been increasing worldwide, and has sometimes led to irreversible impairment of gastrointestinal structure and function. In the present study, we successfully isolated a new phylloketal derivative, deacetylphylloketal (**1**) along with four known compounds from the sponge genus *Phyllospongia.* The anti-inflammatory properties of deacetylphylloketal (**1**) and phyllohemiketal A (**2**) were evaluated using an in vitro co-culture system that resembles the intestinal epithelial environment. A co-culture system was established that consisted of human epithelial Caco-2 cells and phorbol 12-myristate 13-acetate (PMA)-differentiated THP-1 macrophage cells. The treatment of co-cultured THP-1 cells with compounds **1** or **2** significantly suppressed the production and/or gene expression of lipopolysaccharide (LPS)-induced nitric oxide (NO), prostaglandin E2 (PGE2), Interleukin-6 (IL-6), IL-1β and Tumor Necrosis Factor alpha (TNF-α). The expressions of inducible nitric oxide synthase (iNOS), cyclooxygenase (COX)-2 were down-regulated in response to inhibition of NF-kB translocation into the nucleus in cells. In addition, we observed that **1** and **2** markedly promoted the nuclear translocation of Nrf2 and subsequent increase in the expression of heme oxygernase (HO)-1. These findings suggest the potential use of sponge genus *Phyllospongia* and its metabolites as a pharmaceutical aid in the treatment of inflammation-related diseases including IBD.

## 1. Introduction

Inflammatory bowel disease (IBD) that clinically includes Crohn’s disease (CD) and ulcerative colitis (UC) is known to result from inappropriate and chronic inflammatory responses to commensal flora in a genetically susceptible host [1]. In patients with Crohn’s disease, one of the clinical features is discontinuous segments of inflammation that are found in any part of the gastrointestinal tract. The microscopic features of CD include thickened submucosa, transmural inflammation, non-caseating granulomas, strictures and fistulas that lead to bowel obstruction [1,2]. On the other hand, UC is characterized by the limited inflammatory region which usually begins in the rectum and spreads in a continuous fashion.

Numerous cells are involved in maintenance of the structure and function of intestinal tissue. These cells are communicating and strongly influenced by each other. Intestinal epithelial cells, the main player of the cells, are firmly connected by tight junctions to form a semi-permeable barrier. The weakened and compromised epithelial barrier in IBD results in the increased permeability of intestinal mucosa [3]. The disturbance of intestinal homeostasis including weakened epithelial barrier, aberrant immune responses and subsequent exaggerated production of inflammation mediators is believed to associate with the development of IBD [4].

Mono-cultivation of cells has a limitation to mimic the complexity of the mucosal immune system in vitro. Co-cultivation of more than one type of cells make it possible to understand the effects of soluble substance as well as the mechanisms involved in direct contact [5]. For understanding intestinal pharmacology, various in vitro co-culture systems consisting of epithelial cells and immune cells have been developed. Recently, the more sophisticated intestinal co-culture model that mimics the complex structure defining the intestine has been advanced. The in vitro model of intestine is produced by combining human epithelial Caco-2 and differentiated THP-1 macrophages [6]. Caco-2 cell is known to offer transport and permeability characteristics similar to human intestinal tissue [7,8]. This cell-line derived co-culture model provides easy accessibility, handling, and maintenance of cells.

Marine organisms have been paid much attention as a source of structurally diverse and biologically active compounds for the treatment of human diseases [9]. Among them, *Phyllospongia*, one of the most common marine sponge genera in tropical and subtropical areas, has been extensively studied and a total of 132 various secondary metabolites had been isolated and characterized by 2016 [10]. In terms of their chemical structures, the main constituents derived from *Phyllospongia* sponges are sesterterpenoids, especially scalaranes [11], which are classified as C25 (scalarane), C26 (homoscalarane), and C27 (bishomoscalarane) [12]. Bioassay results indicated that some chemicals have pronounced biological activities including antibacterial, antifungal, antileukemic and cytotoxicity [10,13,14,15]. In our search for intestinal anti-inflammatory compounds from marine sponges, the methanolic extract of marine sponge, the genus *Phyllospongia* showed significant inhibitory activity on nitric oxide (NO) production in co-culture system of Caco-2 and THP-1 cells. By bioactivity-guided fractionation, deaceylphylloketal (**1**) and phyllohemiketal A (**2**), showing inhibitory activity, were successfully isolated by Sephadex LH-20 and reversed phase silica column chromatography. Previous studies have afforded several secondary metabolites from the sponge genus *Phyllospongia*. Terpenes (phyllolactones A–E, phyllolactones A–E) and scalarane sesterterpenes (phyllospongins A–E) have been isolated from the organic fraction of *P. lamellosa* collected in Red Sea [12,16]. However, anti-inflammatory potential of sponge genus *Phyllospongia* or its-derived metabolites have not been extensively explored. In our in vitro system, **1** and **2** isolated from sponge genus *Phyllospongia* effectively reduced the production of inflammatory cytokines without cytotoxicity in the concentration tested. Therefore, in the present study, we attempted to evaluate the pharmacological potential of **1** and **2** in the treatment of IBD using co-cultured human Caco-2 and THP-1 macrophage cells.

## 2. Results and Discussion

### 2.1. Isolation of Anti-Inflammatory Scalarane Sesterterpenes from Sponge Genus *Phyllospongia* Using Bioactivity-Guided Fractionation

**Figure 1:**
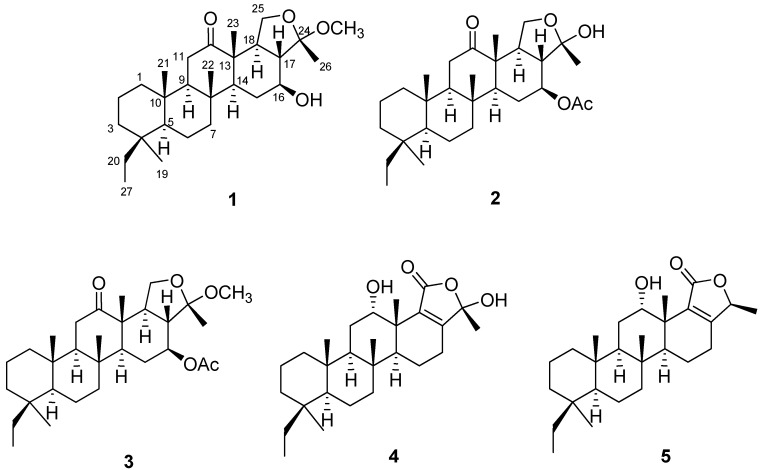
The structures of **1**–**5** isolated from sponge genus *Phyllospongia*.

Deacetylphylloketal (**1**), a new phylloketal derivative, and phyllohemiketal A (**2**) were isolated with phylloketal (**3**), phyllolactone E (**4**) and phyllofolactone B (**5**) from sponge genus *Phyllospongia*. The structures of all isolated compounds were determined by 1D and 2D nuclear magnetic resonance (NMR) experiments based on high-resolution mass spectrometry (HRMS) data (Figure 1). Aside from compound **1**, the four other compounds have been reported in the literature [16,17,18]. Compound **1**, a white powder, was given as the molecular formula of C_28_H_46_O_4_ on the basis of the sodium adduct peak ([M + Na]^+^ = 469.3279, Δ = 3.0 ppm) in the positive QTOF-MS spectrum. The structure of **1** was established by the interpretation of heteronuclear single quantum coherence (HSQC), correlation spectroscopy (COSY), and heteronuclear multiple bond correlation (HMBC) spectra and the spectral data are listed in Table 1. The structure of **1** is very similar to that of **3**, but the conspicuous differences are the absence of a methyl at 2.03 ppm and the upfield chemical shift of H-16 in **1**. This implies the replacement of acetoxy group with hydroxy group at the C-16 position of **1**. Accordingly, **1** was designated as deacetylphylloketal.

### 2.2. The Anti-Inflammatory Effects of ***1**–**5*** in RAW264.7 and THP-1 Macrophages

Initially, anti-inflammatory activities of the isolated compounds **1**–**5** were screened using lipopolysaccharide (LPS)-activated RAW264.7 and THP-1 macrophage cells. In RAW264.7 cell, prior to evaluating anti-inflammatory activities of **1**–**5**, the cytotoxicities of the compounds were measured using CCK-8 assay. Compounds **4** and **5** which possess lactone group in E ring showed a significant cytotoxicity at the concentration range of 1–20 μM while the viability of **1**–**3**-treated cells was found to be similar to that of the non-treated controls (>98% of control, data not shown). Hence, compounds **1**–**3** were employed in further study. To evaluate the inhibitory activity of **1**–**3** on NO production, RAW264.7 cells were treated with each compound **1**–**3** (20 μM) for 1 h and then treated with LPS (1 μg/mL). After 24 h of LPS challenge, the content of NO that is released into culture medium was detected using the Griess reagent. The pretreatment of **1** or **2** led to a significant decrease in LPS-induced NO production. The production of NO was decreased to the level of 35.91, 27.36% and 79.07% of LPS-only treated cells, by the treatment of **1**, **2** and **3**, respectively.

**Figure 2:**
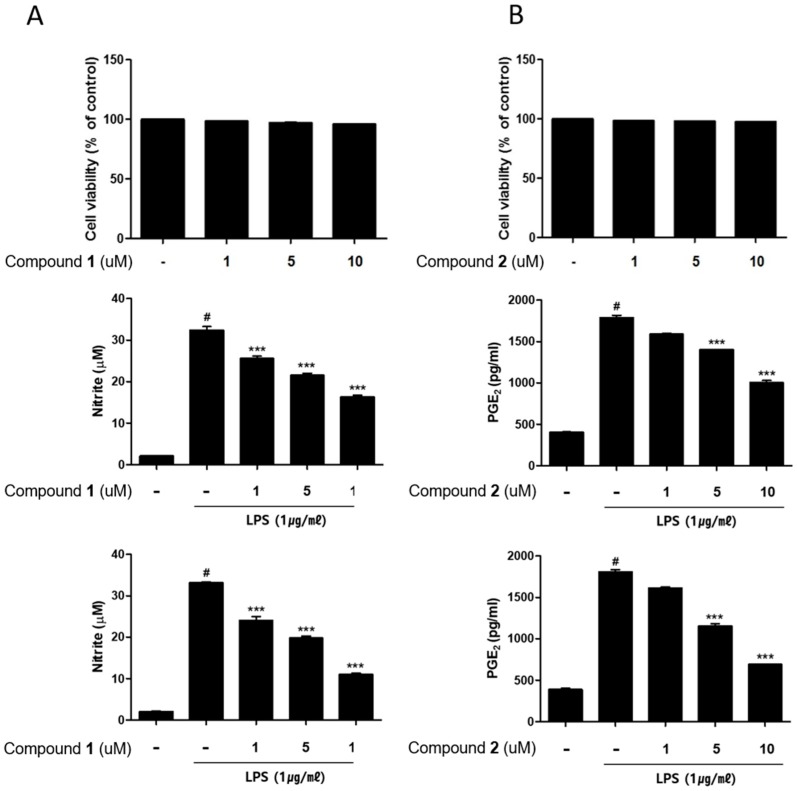
(**A**) Deacetylphylloketal (**1**) and (**B**) phyllohemikal A (**2**) inhibited the production of nitric oxide (NO) and prostaglandin E2 (PGE2) in THP-1 cells without cytotoxicity. THP-1 macrophages were challenged with various concentration of **1** or **2** (1, 5, and 10 μM) without lipopolysaccharide (LPS, for cytotoxicity) or with LPS (for NO and PGE2 production) for 24 h. Results are presented as the means ± standard deviations (SDs) of triplicate experiments; ^#^ *p* < 0.001 compared to LPS-non-treated cells, *** *p* < 0.001 compared to LPS-treated cells.

Anti-inflammatory activities of bioactive compounds **1** and **2** were further investigated in human origin macrophages, THP-1 cells. To measure the cytotoxicity of **1** and **2**, THP-1 macrophages that is differentiated by phorbol 12-myristate 13-acetate (PMA) were challenged with various concentrations of **1** or **2** (1, 5, 10 μM) for 24 h. Compounds **1** or **2** showed no cytotoxicity at the concentration range tested. To measure inhibitory effects of the compounds on NO and prostaglandin E2 (PGE2) production, THP-1 cells were challenged with compounds for 1h followed by LPS (1 μg/mL) was treated. As a result, the pretreatment of cells with **1** or **2** for 24 h led to a significant decrease in LPS-induced NO and PGE2 production (Figure 2).

### 2.3. The Effects of Deacetylphylloketal and Phyllohemikal A on the Pro-Inflammatory Protein Expression and MAPK Phosphorylation in Caco-2 Co-Cultured THP-1 Macrophages

**Figure 3:**
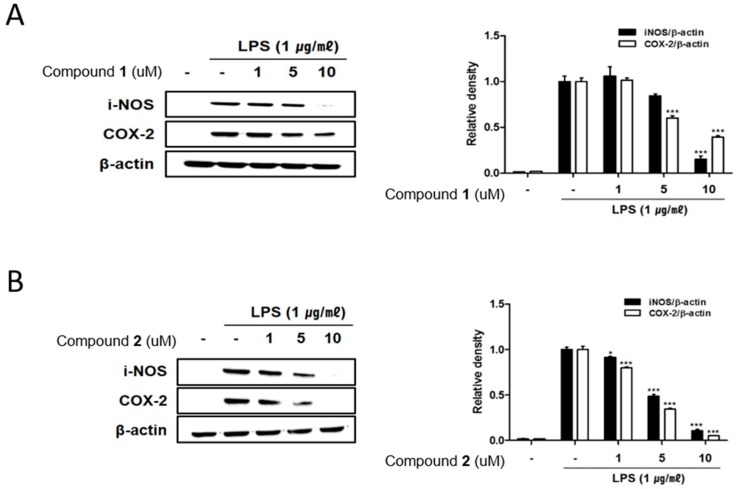
Deacetylphylloketal (**1**) and phyllohemikal A (**2**) down-regulated the expression of inducible nitric oxide synthase (iNOS) and cyclooxygenase (COX-2), decreased the phosphorylation of mitogen-activated protein kinase (MAPK) in a co-culture system of Caco-2 and THP-1 macrophage cells. **1** or **2** (1, 5, and 10 μM) were individually added into the apical compartment of the Caco-2/THP-1 co-culture model. After 1 h, 1 μg/mL LPS was added to the basolateral compartment and cells were incubated overnight. After 24 h of incubation, the expression of iNOS and COX-2 in cells treated with **1** (**A**) and **2** (**B**) were analyzed by Western blot. The representative blot is shown (left panel) and calculated intensities are shown (right panel) after normalization to β-actin. Results are presented as the means ± SDs of triplicate experiments; * *p* < 0.05 and *** *p* < 0.001 compared to LPS-treated cells.

**Figure 4:**
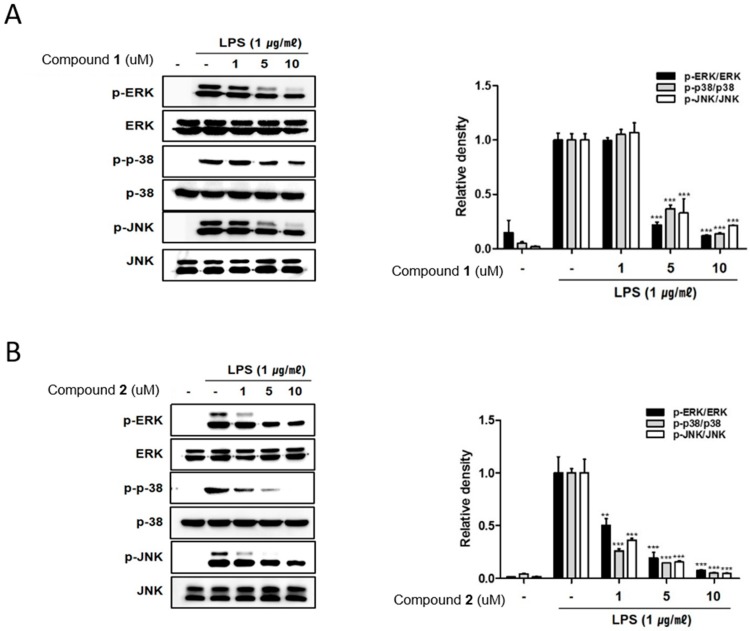
Deacetylphylloketal (**1**) and phyllohemikal A (**2**) decreased the phosphorylation of MAPK in a co-culture system of Caco-2 and THP-1 macrophage cells. **1** or **2** (1, 5, and 10 μM) were individually added into the apical compartment of the Caco-2/THP-1 co-culture model. After 1 h, 1 μg/mL LPS was added to the basolateral compartment and cells were incubated overnight. After 15–30 min of LPS challenge, phosphorylation level of MAPKs (Extracellular signal-Regulated Kinase [ERK], p38, JNK) in cells treated with **1** (**A**) and **2** (**B**) were detected by Western blot. The representative blot is shown (left panel) and calculated intensities are shown (right panel) after normalization to β-actin. Results are presented as the means ± SDs of triplicate experiments; ** *p* < 0.01 and *** *p* < 0.001 compared to LPS-treated cells.

The remarkable increase in the expression of pro-inflammatory proteins such as iNOS and COX-2 are observed in the pathogenesis of colitis [19,20]. In inflammation process, the overexpressed iNOS and COX-2 triggers the production of NO and PGE2 in the activated macrophages [21,22]. To identify the potential of deacetylphylloketal (**1**) and phyllohemikal A (**2**) in regulating inflammation in in vitro co-culture system stimulating the intestine, the expressions of iNOS and COX-2 were evaluated using Western blot analysis. The expression levels of iNOS and COX-2 were up-regulated by LPS treatment, whereas the pretreatment of cells with **1** and **2** (1, 5, 10 μM) significantly attenuated this up-regulation (Figure 3A,B). Next we evaluated the inhibitory effects of these compounds on the phosphorylation of proteins from mitogen-activated protein kinase (MAPK) family including p38, ERK1/2 and JNK. The increased phosphorylation of p38, ERK1/2, and JNK induced by LPS treatment was inhibited by **2** in a concentration of 1 uM (Figure 4B) while the significant inhibition by **1** was observed only at the concentrations from 5 μM upwards (Figure 4A).

### 2.4. The Effects of Deacetylphylloketal and Phyllohemikal A on the Production and mRNA Expression of Proinflammatory Cytokines in Caco-2 Co-Cultured THP-1 Macrophages

**Figure 5:**
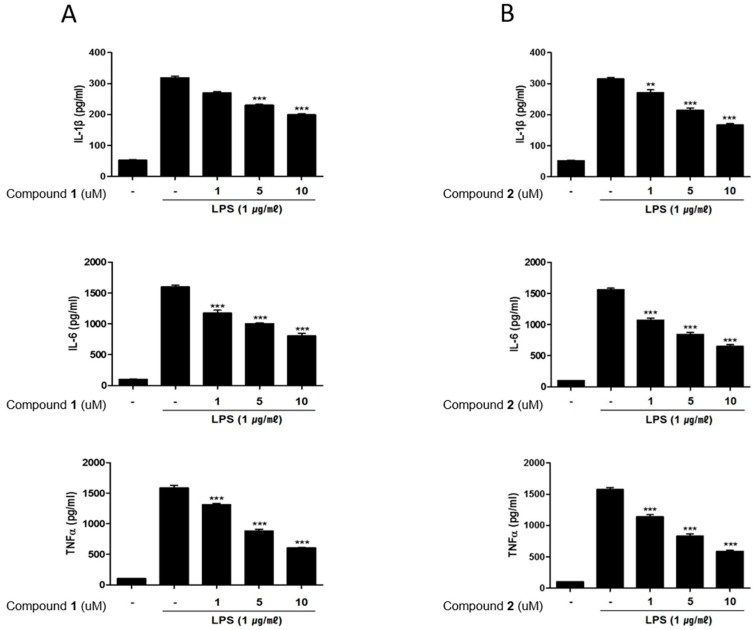
Deacetylphylloketal (**1**) and phyllohemikal A (**2**) reduces the production of inflammatory cytokines in the co-culture system of Caco-2 and THP-1 macrophage cells. Caco-2 cells were incubated with THP-1 macrophages in the presence of various concentrations of **1** or **2** (1, 5, and 10 μM) that were applied to the apical side of this co-culture system. After 1 h, 1 μg/mL LPS was added to the basolateral side and the system was incubated overnight. The levels of Interleukin-1β (IL-1β), IL-6, Tumor Necrosis Factor alpha (TNF-α) in cells treated with **1** (**A**) and **2** (**B**) were subsequently measured from collected culture medium samples using commercially available enzyme-linked immunosorbent assay (ELISA) kits. Results are presented as the means ± SDs of triplicate experiments; ** *p* < 0.01 and *** *p* < 0.001 compared to LPS-treated cells.

**Figure 6:**
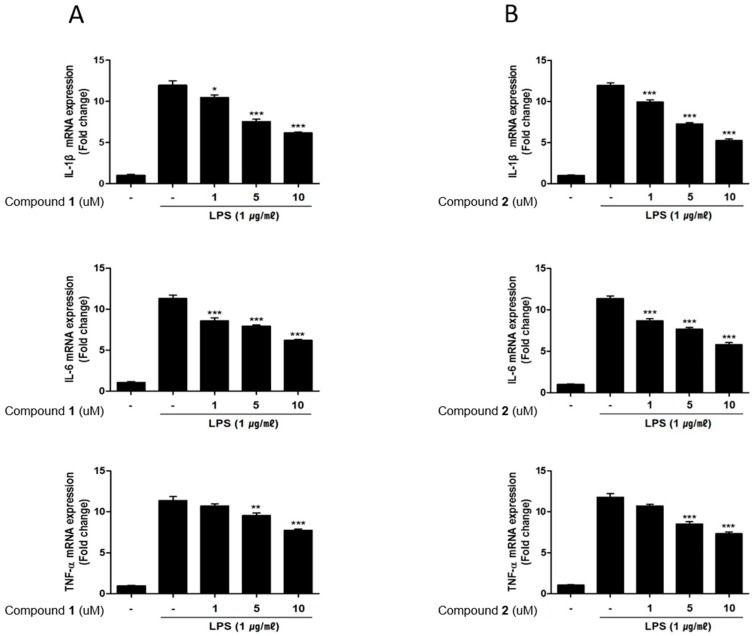
Deacetylphylloketal (**1**) and phyllohemikal A (**2**) attenuated mRNA expression of inflammatory cytokines in the co-culture system of Caco-2 and THP-1 macrophage cells. Caco-2 cells were incubated with THP-1 macrophages in the presence of various concentrations of **1** or **2** (1, 5, and 10 μM) that were applied to the apical side of this co-culture system. After 1 h, 1 μg/mL of LPS was added to the basolateral side and the system was incubated overnight. The mRNA levels of IL-1β, IL-6, TNF-α expressed in cells treated with **1** (**A**) and **2** (**B**) were analyzed using real-time quantitative polymerase chain reaction (qRT-PCR). The quantified expression of each mediator with normalization to glyceraldehydes-3-phosphate dehydrogenase (GAPDH) was presented. LPS, lipopolysaccharide; GAPDH, glyceraldehydes-3-phosphate dehydrogenase. Results are presented as the means ± SDs of triplicate experiments; * *p* < 0.05, ** *p* < 0.01 and *** *p* < 0.001 compared to LPS-treated cells.

It has been demonstrated that immune cells isolated from IBD patients display markedly increased level of inflammatory mediators compared to those from normal tissues [23]. Indeed, increased levels of pro-inflammatory cytokines such as TNF-α, IL-1β, IL-6 are observed in IBD inflammation [24]. Correspondingly, the inhibitors for these cytokines have been proposed to provide an effective treatment strategy for IBD [25]. Therefore, in this study, we measured the effects of **1** and **2** on the production of these inflammatory mediators using enzyme-linked immunosorbent assay (ELISA). Similar to NO and PGE2 levels, the increased levels of IL-1β, IL-6 and TNF-α induced by LPS treatment were decreased by pretreatment of **1** or **2** (1, 5, 10 μM) (Figure 5A,B). The effects of **1** and **2** on mRNA levels of the cytokines were analyzed using PCR. The LPS-induced mRNA expressions of IL-1β, IL-6 and TNF-α were downregulated by pretreatment with **1** or **2** (Figure 6A,B).

### 2.5. The Effects of Deacetylphylloketal and Phyllohemikal A on the Nucleus Translocation of NF-κB and Its Inhibitor, IκB-α

**Figure 7:**
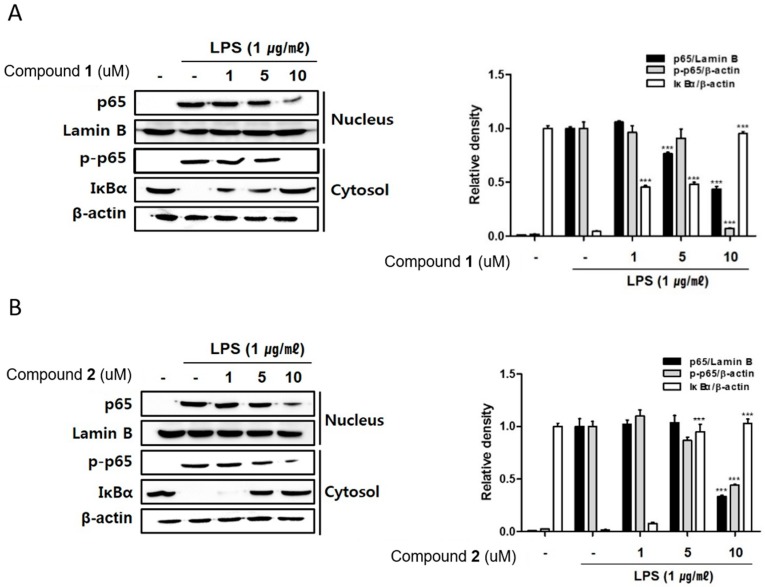
Deacetylphylloketal (**1**) and phyllohemikal A (**2**) inhibited NF-kB translocation into the nucleus in a co-culture system of Caco-2 and THP-1 macrophage cells. **1** or **2** (1, 5, and 10 μM) were individually added into the apical compartment of the Caco-2/THP-1 co-culture model. After 1 h, 1 μg/mL LPS was added to the basolateral compartment and cells were incubated overnight. Cell extracts were biochemically separated into nuclear and cytoplasmic fractions, and the expression of p65, phosphorylated-p65, Lamin B and IκBα in cells treated with **1** (**A**) or **2** (**B**) were analyzed by Western blot. The representative blots are shown (left panels) and calculated intensities are shown (right panels) after normalization to Lamin B (nucleus) or β-actin (cytosol). Results are presented as the means ± SDs of triplicate experiments; *** *p* < 0.001 compared to LPS-treated cells.

The NF-κB transcription factor is known to play key roles in immune and inflammatory signaling via controlling the expressions of the related genes [26,27]. NF-kB binds to proximal promoter of the COX-2 and TNF-α gene, hence the inhibition of DNA-binding activity of NF-κB corresponds to the expressional suppression of inflammatory mediators, iNOS, COX-2 and TNF-α in macrophages [28,29,30]. In an inactivated cell, the NF-κB complex comprising subunits p50 and p65 is localized in the cytoplasm combined with IκB-α, an in hibitor of NF-kB. When cells are activated, IκB-α is phosphorylated by IkB kinase (IKK) complex and dissociated from NF-κB which allows the translocation of NF-κB into the nucleus. The interaction of NF-kB p65 with a specific set of genes promotes subsequent transcription and expression of inflammatory mediators in macrophages [27]. Based on the inhibitory effects of **1** and **2** on the expressions of pro-inflammatory cytokines and proteins, the regulatory effects of these compounds on localization of p65 and phosphorylrated p65 in cell nucleus and cytoplasm have been observed. As shown in Figure 7, Western blot analysis showed that the treatment of **1** and **2** (1, 5, 10 μM) inhibited the phosphorylation of p65 in cytoplasm and also attenuated the translocation of p65 into nuclei. The inhibition of p65 phosphorylation in cytoplasmic fraction was significant by **1** or **2** at the concentration 10 μM (Figure 7A,B). Intriguingly, pretreatment of **1** and **2** significantly up-regulated total level of IκB-α protein in cytoplasm, which may account for the enhanced binding of IκB-α to p65 which may prevent the accessibility of NF-κB into nucleus to bind the promoter. At the concentration of 1 μM, the increased expression of cytoplasmic IκB-α was observed by the treatment of **1** while no significant change was induced by **2** (Figure 7A,B). Our results provide evidence that **1** and **2** disrupt interactions of p65 subunit of NF-kB with a specific sets of target genes results in the downregulate of the subsequent transcription and expression of pro-inflammatory mediators [31,32].

### 2.6. The Effects of Deacetylphylloketal and Phyllohemikal A on the Expression of HO-1 Mediated by Nuclear Translocation Nrf2 in Caco-2 Co-Cultured THP-1 Macrophages

**Figure 8:**
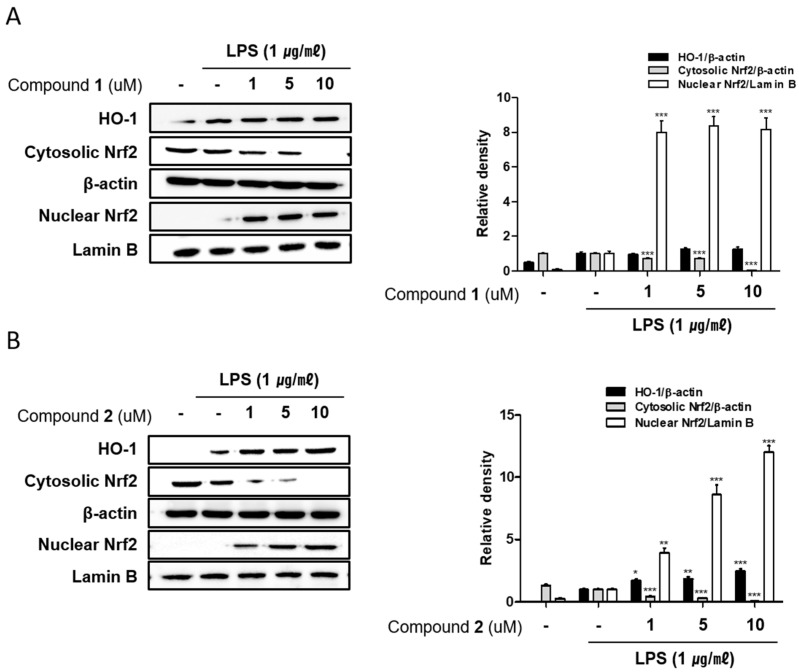
Deacetylphylloketal (**1**) and phyllohemikal A (**2**) increased HO-1 expression and induced the nuclear translocation of Nrf2 in a co-culture system of Caco-2 and THP-1 macrophage cells. **1** or **2** (1, 5, and 10 μM) were individually added into the apical compartment of the Caco-2/THP-1 co-culture model. After 1 h, 1 μg/mL LPS was added to the basolateral compartment. Following LPS challenge of 12 h and 1h, the expression of HO-1 and Nrf2 in cells treated with **1** (**A**) or **2** (**B**) were analyzed by Western blot, respectively. The representative blot is shown (left panel) and calculated intensities are shown (right panel) after normalization to Lamin B (nucleus) or β-actin (total protein or cytosol). Results are presented as the means ± SDs of triplicate experiments; * *p* < 0.05, ** *p* < 0.01 and *** *p* < 0.001 compared to LPS-treated cells.

**Figure 9:**
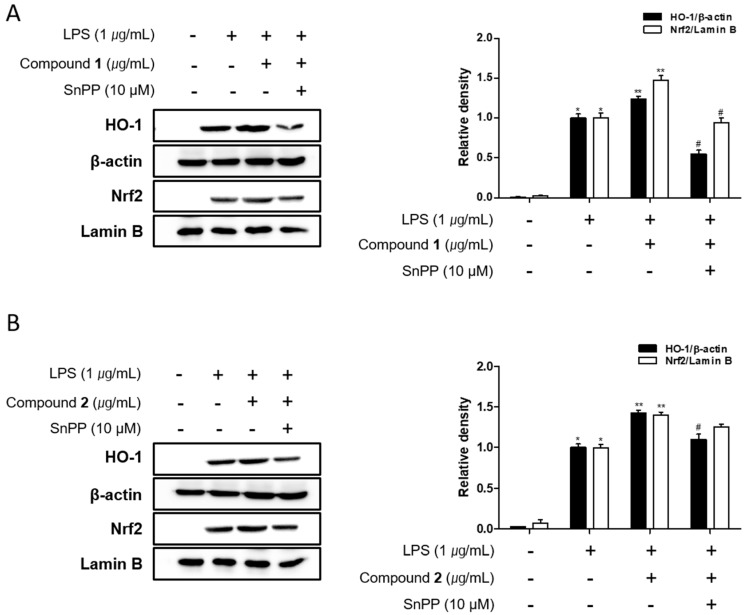
Deacetylphylloketal (**1**) and phyllohemikal A (**2**) increased HO-1 expression and induced the nuclear translocation of Nrf2 in a co-culture system of Caco-2 and THP-1 macrophage cells. **1** or **2** (10 μM) were individually added into the apical compartment of the Caco-2/THP-1 co-culture model in the absence or presence of SnPP, a HO-1 inhibitor. The expression of HO-1 and Nrf2 in cells treated with **1** (**A**) or **2** (**B**) were analyzed by Western blot after LPS challenge of 12 h and 1h, respectively. The representative blot is shown (left panel) and calculated intensities are shown (right panel) after normalization to Lamin B (Nrf2) or β-actin (HO-1). Results are presented as the means ± SDs of triplicate experiments; * *p* < 0.05 and ** *p* < 0.01 compared to cell treated with LPS only, ^#^ *p* < 0.05 compared to cell treated with LPS and compound.

It has been reported that family proteins from MAPK have been associated with Nrf-2/HO-1 pathway in an inducer- and cell type-dependent manner. HO-1 is known to suppress the expression of pro-inflammatory proteins and cytokines in the activated macrophages [33,34]. Recently, the important role of nuclear factor [erythroid-derived 2]-like 2 (Nrf-2)/HO-1 signaling as anti-inflammatory and anti-oxidant effectors has been demonstrated in animal model of IBD. In a colitis mouse model induced by dextran sulphate sodium (DSS), the markedly enhanced expression of Nrf-2/HO-1 was detected under colitis wound repair [35]. In this model, oral administration of 6-shogal suppressed pro-inflammatory cytokines, TNF-α, IL-6 and IL-1β and promoted the expressions of Nrf-2 and HO-1 which results in acceleration of colitis wound repair. Also, in colitis mice model induced by 2,4,6-trinitrobenzenesulfonic acid (TNBS), it has been revealed that the administration of lyophilised microalgal biomass prepared from *Chlamydomonas debaryana* increased the expressions of Nrf-2 and HO-1 [36]. To identify if **1** and **2** can regulate Nrf-2/HO-1 signaling, Western blot analysis was conducted. As shown in Figure 8, the protein level of HO-1 in Caco-2 co-cultured THP-1 cells were increased by the treatment of **1** or **2** at the concentration tested (1, 5, 10 μM). Nrf-2 is a master regulator of antioxidant enzymes including HO-1 via binding to antioxidant response element (ARE). Following **1** or **2** exposure, the levels of nuclear Nrf2 was sharply increased with a concomitant decrease in the cytoplasmic levels. The induction of HO-1 expression and nuclear translocation of Nrf2 by **1** or **2** treatment was attenuated by Snpp, a HO-1 inhibitor (Figure 9). In the present study, we observed that **1** and **2** markedly promoted the nuclear translocation of Nrf2 and subsequent increase in the expression of HO-1.

## 3. Materials and Methods

### 3.1. Materials

The LPS (*Escherichia coli* 0127:B8), dimethyl sulfoxide (DMSO), PMA (phorbol 12-myristate 13-acetate) were purchased from Sigma-Aldrich (St, Louis, MO, USA). MEM medium, RPMI 1640 medium, fetal bovine serum (FBS), penicillin/streptomycin and phosphate-buffered saline (PBS) were purchased from Gibco Life Technologies (Grand Island, NY, USA). The Griess Reagent System was obtained from promega (Promega, Madison, WI, USA). Enzyme-linked immunosorbent assay (ELISA) kits for TNF-α, IL-6, and PGE2 were purchased from R&D Systems (Minneapolis, MN, USA). IL-1β, TNF-α, IL-6 and GAPDH primers were purchased from Bioneer (Daejeon, Korea). Tin protoporphyrin IX (SnPP) was purchased from Porphyrin Products (Logan, UT, USA). Primary antibodies, including iNOS, COX-2, p65, Lamin B, phospho-p65, IκBα, phospho-ERK, ERK, phospho-p38, p38, phospho-JNK, JNK, HO-1 and Nrf2 were purchased from Cell Signaling Technology, Inc. (Beverly, MA, USA). Primary monoclonal anti-β-Actin antibody was purchased from Sigma-Aldrich (St, Louis, MO, USA). The secondary antibody was purchased from Jackson ImmunoResearch Laboratories, Inc.

### 3.2. Isolation of Scalarane Sesterterpenes from Sponge Genus *Phyllopspongia*

Specimen of marine sponge, genus *Phyllospongia* was collected in Cebu, the Philippines, in August, 2006. The samples were identified by Dr. Ji-Hyun Kim and a voucher (06KNU-PIL-6) was deposited in the Marine Biodiversity Institute of Korea (MABIK). The freeze-dried specimen (0.8 Kg) was extracted with MeOH (2 L) for 24 h twice at room temperature and partitioned between H_2_O and CH_2_Cl_2_ for removing salts. The organic layer was repartitioned between 85% aqueous MeOH and hexane and then the residue from the polar layer (4 g) was subjected to a reversed phase column chromatography, eluting from 50% H_2_O to 100% MeOH stepwise with 10% MeOH increase to produce the six fractions (I-VI). Fraction VI (1 g), showing the NO inhibitory activity, was again chromatographed on Sephadex LH-20 eluted with 100% MeOH to yield five subfractions (M1-M5), Following this separation, the bioactive subfraction M5 (300 mg) was separated by silica-phase high-performance liquid chromatography (HPLC) to obtain five impure compounds at the retention time 12–18 min and 33 min, respectively. The HPLC separation was conducted under a condition of YMC silica (250 × 10 mm) column, isocratic elution with mixed solvents of 40% EtOAc and 60% Hexane at flow rate 2.0 mL/min. Each mixture was purified by reversed phased HPLC [YMC ODS C18 (250 × 10 mm) column, elution solvent of 95% ACN and 5% H_2_O, and flow rate of 2.0 ml/min] to yield pure deacetylphylloketal (**1**, 4.2 mg), phyllohemiketal A (**2**, 6.5 mg), phylloketal (**3**, 3.2 mg), phyllolactone E (**4**, 5.0 mg) and phyllofolactone B (**5**, 5.1 mg).

### 3.3. Cell Cultures

The murine macrophage cell line (RAW264.7) were purchased from Korea Cell Line Bank (Seoul, Korea). The cell line was maintained in DMEM containing 20 mM HEPES, 2 mM L-Glutamine, 10% FBS with penicillin (100 IU/mL) and streptomycin (10 mg/mL) at 37 °C in a humidified atmosphere of 95% air-5% CO_2_. RAW264.7 cells were cultured in DMEM (Gibco BRL, Grand Island, NY, USA) containing 20% (v/v) FBS and 1% (v/v) antibiotics (100 U/mL penicillin and 100 μg/mL streptomycin). The human epithelial cell line (Caco-2) and human monocytic cell line (THP-1) were obtained from the American Type Culture Collection (ATCC, Manassas, VA, USA). The Caco-2 cells were cultured in Eagle’s minimum essential medium (EMEM) (Gibco BRL, Grand Island, NY, USA) containing 20% (v/v) FBS and 1% (v/v) antibiotics (100 U/mL penicillin and 100 μg/mL streptomycin). THP-1 cells were cultured in RPMI1640 (Gibco BRL, Grand Island, NY, USA) containing 10% (v/v) FBS, 1% (v/v) antibiotics (100 U/mL penicillin and 100 μg/mL streptomycin) and 0.05 mM 2-Mercaptoethanol. The cells were incubated in a humidified atmosphere of 95% air-5% CO_2_. After that, THP-1 cells were treated with PMA for macrophage differentiation as below.

### 3.4. Differentiation of THP-1 to Macrophages

THP-1 cells were differentiated by incubation in 50 nM of PMA (2-Mercaptoethanol, phorbol 12-myristate 12-acetate) for three days followed by incubated in fresh medium without PMA for additional two days [37].

### 3.5. In Vitro Co-Culture Model of Intestine

For establishment of co-culture system of human intestine, Caco-2 cells were seeded at 3.75 × 10^5^ cells/well on transwell inserts (0.4 μm pore size, Corning CoStar Corp., Cambridge, MA, USA) and maintained in growth media EMEM described in 3.3 for 14–20 days in an incubator at 37 °C with a 5% CO_2_ atmosphere. Culture medium was changed every 3 days until the cells were fully differentiated (Transepithelial/transendothelial electrical resistance [TEER] value > 1200 Ω cm^2^). THP-1 cells were independently seeded at 8.5 × 10^6^ cells into the bottom plate of 6-well transwell plate in growth media RPMI1640 described in Section 3.3. Then, the insert that is polarized with Caco-2 monolayer was added. The upper chamber will reflect the apical side and the lower chamber the basolateral side of the intestinal epithelium [5]. For evaluation of anti-inflammatory effects of deacetylphylloketal and phyllohemikal A, 1 μg/mL of LPS was added on the basolateral compartment of plate and then the compound to be tested was added at the concentration indicated to the apical compartment of plate. After 24 h incubation, the contents of nitrite and inflammatory mediators produced within culture supernatant from the basolateral side were determined.

### 3.6. TEER Measurement

TEER value measurements were performed to test monolayer integrity using a Millicell-ERS (Millipore, MA, USA). The TEER was calculated as following: TEER (Ω cm^2^) = Resistance − Blank resistance (Ω) × Membrane surface Area (cm^2^).

### 3.7. Cell Viability Assay

Cell viability was measured using the CCK-8 (Cell Counting Kit-8, #CK04, Dongjindo laboratory, Japan), according to the manufacturer’s instructions. Briefly, THP-1 cells were seeded at 1 × 10^4^ cells/well in 96-well plates and incubated for 24 h. Then, the cells were treated with deacetylphylloketal or phyllohemikal A of various concentrations for 24 h. After incubation, 10 μL of CCK-8 solution was added to each well and incubated for 3 h at 37 °C The absorbance was measured using a microplate reader (Bio-Tek Company, Winooski, VT, USA) at 450 nm. The experiments were performed in triplicate.

### 3.8. Measurement of NO and PGE2 Production

THP-1 cells were seeded at 1 × 10^4^ cells/well in 96-well plates and incubated for 24 h. Then, the cells were treated with deacetylphylloketal or phyllohemikal A of various concentrations for 24 h. The level of NO produced in culture medium was determined by Griess reagent (Promega, Madison, WI, USA). Griess assay is one of the most common methods for quantifying NO in culture medium which detects nitrite that is produced from NO in oxygenated media due to NO’s high reactivity [38]. The 100 μL of culture supernatants was mixed with 50 μL of sulfanilamide solution and 50 μL of N-1 napthylethylenediamine dihydrophloride (NED) solution. And then incubated for 10 min at room temperature, protected from light. After 15 min of incubation, the optical density was determined at 540 nm with a microplate reader (Bio-Tek Company, Winooski, VT, USA). The concentration of PGE2 was quantified using an ELISA kit (R&D System, Minneapolis, MN, USA) according to the manufacturer’s instructions. The culture medium as applied in ELISA assay without dilution.

### 3.9. Measurement of IL-1β, TNF-α and IL-6 Production

The concentration of IL-1β, TNF-α and IL-6 that is produced within culture supernatant in co-culture system was quantified using an ELISA kit (R&D System, Minneapolis, MN, USA) according to the manufacturer’s instructions. Twenty-four hours after the treatment of test compounds and LPS, the culture medium was collected from the basolateral compartment of transwell plate and applied in ELISA assay without dilution. The absorbance was measured at 450 nm using a microreader (Biotek Instruments, Inc., Winooski, VT, USA).

### 3.10. Real-Time Quantitative Polymerase Chain Reaction (qRT-PCR)

Twenty-four hours after the treatment of test compounds and LPS, total RNA was isolated from THP-1 cells incubated in co-culture system using Trizol reagent (Invitrogen, Carlsbad, CA, USA). Reverse transcription of mRNA into cDNA was performed with QuantiTect Reverse Transcription Kit (Qiagen, Valencia, CA, USA) according to the manufacturer’s instructions. Quantification was performed by a Mx3005P QPCR Systems (Agilent Technologies, Santa Clara, CA, USA) by using GoTaq qPCR Master Mix (Promega, Mannheim, Germany) with specific primers. qRT-PCR reactions were performed initial denaturation at 95 °C for 2 min followed by 40 cycles of 95 °C for 15 s and 60 °C for 60 s. The data of each gene was normalized to the housekeeping gene GAPDH.

### 3.11. Western Blot

Protein isolated form THP-1 cells incubated in co-culture system was applied to Western blot assay. After incubation of cells with test compounds and LPS as indicated, THP-1 cells were washed three times with ice-PBS and extracted with Radioimmunoprecipitation assay (RIPA) buffer containing a protease inhibitor cocktail (Santa Cruz, CA, USA) for 40 min on ice. Protein lysates were centrifuged at 13,000× *g* for 30 min at 4 °C. Thirty μg of the lysed proteins quantified by Bradford assay were separated on sodium dodecyl sulfate polyacrylamide gel electrophoresis (SDS-PAGE, 8–12%) at 100 V and transferred to PVDF membrane. The membranes were blocked with 5% nonfat milk in PBST buffer for 1h at room temperature. The membranes were incubated with 1:1000 diluted primary antibodies (Cell Signaling Technology, Inc., Danvers, MA, USA) at 4 °C overnight. The membranes were washed three times with PBST buffer followed by incubated with 1:10000 diluted secondary antibodies for 1 h at room temperature. Bands were visualized using enhanced chemiluminescence (ECL) solution (Thermo Scientific) and calibrated by the Chemidoc Imaging System (Bio-Rad; Hercules, CA, USA). The density value of the protein bands was normalized to Lamin B (nuclear) or β-actin (total protein or cytosol).

### 3.12. Statistical Analysis

All data were through Shapiro-wilk normality test and Levene’s equality of variance test by SPSS 17.0 program (SPSS Inc., Chicago, IL, USA). Then, the results were analyzed and converted to graph using Prism version 5.00 software (GraphPad Software, San Diego, CA, USA); one-way analyses of variance (ANOVA) were used for parametric data or Kruskal-Wallis for non-parametric data with Dunnett or Dunns post hoc tests, respectively. Statistical significance is indicated by *p* < 0.05. Data were expressed as the mean ± standard deviations (SDs) of three independent experiments.

## 4. Conclusions

The co-culture in vitro system that resembled intestinal environment was established by combining epithelial Caco-2 cells and THP-1 macrophage cells. The treatment of **1** and **2** isolated from sponge genus *Phyllospongia* exhibited potent anti-inflammatory activity by regulating the expression levels of pro-inflammatory (TNF-α, IL-6, IL-1β) and anti-inflammatory (Nrf-2 and HO-1) factors. Compounds **1** and **2** down-regulated the expressions of iNOS and COX-2, as well as attenuating nuclear translocation of NF-κB. Considering NF-κB may has been viewed as a master switch regulating inflammation in macrophages, these studies support the potential use of marine sponge, genus *Phyllospongia* as a therapeutic agent in the treatment of IBD.

## Figures and Tables

**Table 1:** The spectral data for **1** in CDCl_3_ (^1^H for 500 MHz, ^13^C for 125 MHz).

No.	^1^H, mult (J Hz)	^13^C	No.	^1^H, mult (J Hz)	^13^C
1	0.79, m; 1.58, m	39.8, t	15	1.48, m	30.2, t
2	1.39, m; 1.49, m	18.1, t		1.95, ddd (12.7, 4.9, 2.5)	
3	0.84, m; 1.67, m	36.4, t	16	3.77, td (10.5, 5.1)	70.1, d
4		36.1, s	17	1.64, dd (12.7, 10.5)	54.8, d
5	0.86, m	58.6, d	18	2.28, m	46.8, d
6	1.47, m; 1.57, m	18.0, t	19	0.80, s	28.5, q
7	0.95, td (12.5, 3.7)	41.9, t	20	1.18, m; 1.53, dd (14.2, 7.6)	24.5, t
	1.83, dt (12.5, 3.2)		21	0.88, s	16.4, q
8		37.6, s	22	1.05, s	16.8, q
9	1.17, dd (13.5, 2.5)	61.3, d	23	1.12, s	13.7, q
10		38.1, s	24		104.4, s
11	2.23, dd (13.5, 2.5); 2.60, t (13.5)	34.8, t	25	3.63, t (8.6); 4.26, t (8.6)	67.5, t
12		215.3, s	26_3_	1.49, s	21.9, q
13		51.4, s	27	0.75, t (7.6)	8.8, q
14	1.28, m	58.7, d	OCH	3.25, s	48.4, q

q = CH_3_, t = CH_2_, d = CH, s = C.

## Abbreviations

Chk2	Checkpoint kinase 2
LPS	lipopolysaccharide
PGE2	Prostaglandin 2
NO	Nitric oxide
iNOS	Inducible nitric oxide synthase
COX	Cyclooxygenase
HO-1	Heme oxygenase-1
NF-kB	Nuclear factor kappa-light-chain-enhancer of activated B cells
Nrf2	Nuclear factor erythroid 2 related factor 2
MAPK	Mitogen-activated protein kinase
HRESIMS	High-resolution electrospray ionization Mass spectrometry
QTOF	Quadrupole time of flight mass spectrometer
HSQC	Heteronuclear single quantum coherence
COSY	Correlation spectroscopy
HMBC	Heteronuclear multiple bond correlation
